# Influence of Laser Power on the Microstructure, Wear Resistance, and Cavitation-Erosion Behavior of Laser-Clad Fe_45_Cr_25_Ni_20_Ti_5_Mo_5_ Multi-Principal-Element Alloy Coatings

**DOI:** 10.3390/ma19183981

**Published:** 2026-09-19

**Authors:** He Bao, Wei Liu, Tuo Wang, Li Fu, Xin Wei, Xiaoming Chen, Xidong Hui

**Affiliations:** 1 School of Materials Science and Engineering, Xinjiang University, Urumqi 830017, China; 2 Water Machinery and Remanufacturing Technology Engineering Laboratory of Zhejiang Province, Hangzhou River Mechanical and Electrical Equipment Engineering Co., Ltd., Hangzhou 310024, China; 3 Key Laboratory of Surface Engineering of Equipment for Hydraulic Engineering of Zhejiang Province, Standard and Quality Control Research Institute, Ministry of Water Resources, Hangzhou 310024, China; 4 State Key Laboratory for Advanced Metals and Materials, University of Science and Technology Beijing, Beijing 100083, China

**Keywords:** laser cladding, multi-principal-element alloy coating, wear resistance, cavitation-erosion resistance

## Abstract

Flow-passing components such as volutes and fixed guide vanes operating in high-velocity liquid flows with high sediment concentrations are subjected to long-term cavitation erosion and sand-particle abrasion, which cause substantial economic losses. To reduce the manufacturing and maintenance costs of mechanical equipment serving such environments, laser cladding was employed to fabricate Fe_45_Cr_25_Ni_20_Ti_5_Mo_5_ multi-principal-element alloy coatings on Q235 steel substrate, and the effect of laser power on the microstructure and properties of the coatings was systematically investigated. On the basis of preliminary investigations, the present work elaborately analyzes the wear resistance and cavitation-erosion resistance of coatings fabricated under laser powers of 1000 W, 1200 W and 1400 W. The results reveal that the coating hardness gradually decreases from a maximum value of 485.67 HV_0.2_ to 363.15 HV_0.2_ with increasing laser power. When the laser power is 1200 W, the coating prepared under this laser power maintains a good balance between hardness and microstructural integrity. Under identical friction-and-wear test conditions, its wear rate reaches only 3.15 × 10^−5^ mm^3^/(N·m), which is reduced by 19.64%, 27.92% and 39.19% compared with the coatings produced at 1000 W, 1400 W and bare Q235 steel, respectively. After a 20h cavitation-erosion test, the mass loss of this coating is merely 2.56 mg, representing reductions of 72.88%, 81.5% and 96.91% relative to the 1000 W coating, 1400 W coating and Q235 steel substrate. The coating fabricated at 1200 W exhibits outstanding wear resistance and cavitation-erosion resistance. The results indicate that, under the experimental conditions of this study, the wear resistance and cavitation-erosion resistance of the coating can be effectively optimized by adjusting the laser power.

## 1. Introduction

Flow-passing components such as volutes and fixed guide vanes serve long term in high-velocity liquid-flow environments that contain abundant cavitation bubbles. High-pressure shock waves and high-speed micro-jets generated by bubble collapse induce fatigue spalling and plastic damage on material surfaces, which is defined as cavitation erosion [1,2]. Meanwhile, these mechanical components are continuously subjected to abrasive damage caused by sediment particles [3,4,5]. Such dual damage mechanisms drastically shorten the service life of these components. Annual economic losses arising from equipment maintenance and replacement due to cavitation erosion and abrasion exceed hundreds of billions of RMB, and equipment safety accidents may even occur under extreme service conditions [6].

Q235 steel is widely adopted for manufacturing mechanical components owing to its excellent weldability and low cost. Nevertheless, its service life is severely restricted by low hardness, poor cavitation resistance and inferior wear resistance. To address this issue, depositing protective coatings on material surfaces has become an effective surface-protection strategy [7,8,9]. As an advanced surface-modification technique, laser cladding shortens the material-design cycle compared with conventional processes such as sintering and melting [10,11]. In comparison with other surface-modification technologies, laser cladding exhibits merits including a narrow heat-affected zone, low dilution rate, minor elemental segregation, a uniform and dense cladding layer, and high metallurgical bonding strength [12]. This technique has been extensively applied to modify the surfaces of Q235-steel-based mechanical components for fabricating cavitation- and wear-resistant coatings. Yang et al. [13] fabricated FeCoCrNiMoSi_x_ (x = 0.5, 1.0, 1.5) coatings with varied silicon contents on Q235 steel substrate via laser cladding. The results revealed that increasing Si content aggravated lattice distortion and promoted the formation of Si-rich intermetallic phases, which remarkably improved the hardness and wear resistance of coatings; consequently, the tribological performance of Q235 substrate was effectively enhanced. The results revealed that increasing Si content aggravated lattice distortion and promoted the formation of Si-rich intermetallic phases, which remarkably improved the hardness and wear resistance of coatings; consequently, the tribological performance of Q235 substrate was effectively enhanced. Yefeng et al. [14] deposited FeCoCrNiB_0.2_ + 20 wt.% WC coatings on Q235 steel substrate by laser cladding. It was found that the coating achieved a hardness of 806 HV, and its cavitation-erosion resistance was substantially superior to those of WC-free FeCoCrNiB_0.2_ coating and bare Q235 steel substrate. Shao et al. [15] prepared Cu-10Al coatings with different mass fractions of MoS on Q235 steel surface using laser cladding. The maximum hardness of the coatings reached 424.56 HV, demonstrating excellent wear resistance.

Multi-principal-element alloys break the conventional alloy design dominated by a single principal component. It refers to a non-equimolar multicomponent system containing three or more principal elements with relatively high atomic fractions of the principal elements. The high mixing-entropy effect effectively suppresses the precipitation of intermetallic compounds and facilitates the formation of stable simple solid-solution structures, delivering distinctive performance advantages over conventional alloys [16,17]. Four core characteristics, namely, a high-entropy effect, severe lattice distortion, sluggish diffusion and a cocktail effect, endow these materials with outstanding strength-toughness, wear resistance and corrosion resistance, making them a research hotspot in the field of laser-cladding surface modification [18,19,20,21,22,23]. Yong et al. [24] synthesized Co-Cr-Fe-Ni-Mo-Si multi-principal-element alloys with excellent high-temperature wear resistance. The FeNiCrMoAl multi-principal-element alloy fabricated by Niu et al. [25] exhibited favorable cavitation-erosion resistance and corrosion resistance. The same research group also developed the Fe_23.7_Co_23.8_Ni_23.8_Cr_23.7_Mo_5_ alloy, which also possessed superior cavitation-erosion and corrosion resistance [26]. Combining laser cladding with multi-principal-element alloys integrates their respective strengths, enabling the fabrication of coatings with further improved comprehensive properties.

To date, few investigations have focused on laser-clad Fe_45_Cr_25_Ni_20_Ti_5_Mo_5_ coatings. As reported by Yong et al. [24], Niu et al. [25], and other listed studies, most existing investigations add only a single element (either Ti or Mo) to the Fe–Cr–Ni system and primarily focus on equiatomic high-entropy alloy coatings. In comparison, the present work develops a non-equiatomic Fe_45_Cr_25_Ni_20_Ti_5_Mo_5_ coating with trace additions of both Ti and Mo elements. The Fe_45_Cr_25_Ni_20_Ti_5_Mo_5_ coating uses Fe, Cr and Ni as the main matrix elements. Among them, Fe acts as the matrix skeleton element to construct the solid-solution system and reduce the preparation cost. Cr serves as a passivating element to improve the corrosion resistance of the coating and induce solid-solution strengthening. Ni stabilizes the FCC solid-solution phase, improves the toughness of the coating and restrains crack propagation. Ti and Mo are used as minor strengthening elements. Ti tends to form precipitates such as TiC to achieve dispersion strengthening, while Mo strengthens the matrix via lattice distortion and optimizes the pitting resistance of the passive film. Systematic studies regarding the effect of laser power on the wear resistance and cavitation-erosion resistance of this specific alloy composition remain limited. In this work, newly developed Fe_45_Cr_25_Ni_20_Ti_5_Mo_5_ multi-principal-element alloy coatings were fabricated on Q235 steel substrate by laser cladding. Friction-and-wear tests as well as ultrasonic cavitation-erosion tests were carried out on the coatings. The influences of different laser powers on its microstructure and cavitation-erosion resistance as well as other properties were explored. This study aims to provide a superior material alternative and experimental–theoretical basis for surface modification of flow-passing components operating under high-velocity liquid-flow conditions.

## 2. Sample Preparation and Experimental Methods

### 2.1. Sample Preparation

Q235 steel was used as the substrate and machined into plates with dimensions of 120 mm × 60 mm × 30 mm. Laser-cladding technology was adopted to fabricate a coating with the same surface area on the plate substrate. Before cladding, the substrate was ground and polished using 600# sandpaper, followed by cleaning with 99.9% industrial alcohol. After the substrate dried, it was preheated at 200 °C for 2 h. Spherical Fe, Cr, Ni, Ti and Mo powders with purity higher than 99.9% were selected. The particle size of all high-purity elemental powders ranged from 45 μm to 135 μm. The chemical composition of the powder is listed in Table 1. The powders were thoroughly mixed for 10 h using an SYH-5L three-dimensional motion mixer manufactured by Shanghai Tongshen Industrial Equipment Co., Ltd. (Shanghai, China) Subsequently, coatings were deposited on the Q235 substrate by coaxial powder-feeding laser-cladding equipment (Model FANUC M2000/25, Nanjing Huarui Optoelectronic Technology Co., Ltd., Nanjing, China). This laser-cladding system adopts a fiber laser with a wavelength of approximately 1070 nm and a spot diameter of 3 mm. During laser cladding, the overlap rate was 50%. Argon was adopted as the shielding gas, and the flow rate of shielding gas was approximately 12 L/min. The carrier gas flow rate was approximately 8 L/min. A rotary disk powder feeder was used. Coatings with different laser powers were prepared according to the process parameters listed in Table 2. The coating thickness was approximately 1 mm and the width was 60 mm. Finally, the molybdenum wire cutting equipment is used to cut the sample into a size suitable for performance testing and observation, in preparation for subsequent experiments.

### 2.2. Microstructural Characterization

The surface and cross-sectional morphologies of the coatings were analyzed using a scanning electron microscope (SEM; Zeiss Supra 55, Oberkochen, Germany). The working distance was 8 mm, and the detector mode was secondary electron. This SEM was equipped with an energy-dispersive X-ray spectrometer (EDS; Zeiss Merlin Compact, Oberkochen, Germany) to characterize the elemental distribution on the surface or cross-section of specimens. The accelerating voltage was set to 30 kV during scanning. The phase composition and phase structure of the coatings were determined using an X-ray diffractometer (XRD, Rigaku SmartLab 9 kW, Akishima, Japan). The scanning angle ranged from 10° to 90°, the scanning speed was 4°/min, and the step size was 0.02°. The operating voltage and current were 40 kV and 40 mA, respectively, and the X-ray source used was Cu Kα radiation.

### 2.3. Performance Testing

A micro-Vickers hardness tester (Shanghai Taiming HXD-1000TMC, Shanghai, China) was employed to measure the hardness of coatings. A load of 1.96 N was applied and held for 10 s during testing. For one specimen prepared at each laser power, three hardness measurements were taken at intervals of 10 μm along the same horizontal direction, and the average value was taken as the hardness of this horizontal position. On one specimen for each laser power, a tribometer (UMT Tribo Lab™, Bruker, Ettlingen, Germany) was used to evaluate the wear resistance under dry-sliding friction conditions. An Si3N4 ball with a diameter of 5 mm served as the counter-body. The test was performed for 30 min under a normal load of 20 N, frequency of 3 Hz, ambient temperature of 25 °C and reciprocating sliding distance of 10 mm, producing a 10 mm long scratch. The friction coefficient data were directly exported by the testing instrument. The wear profiles were quantitatively analyzed using a three-dimensional optical profilometer, and the wear volume was directly output by the instrument. The cavitation-erosion resistance was measured using an ultrasonic cavitation tester (XOQS-1200, Nanjing Xianou, Nanjing, China). The test was conducted in strict accordance with ASTM G32-16 standard [27]. The vibration frequency was 20 kHz and the amplitude was 50 μm, with deionized water kept at a constant temperature of 20 °C. Three specimens were used for parallel cavitation-erosion tests for each laser power, and each specimen was subjected to a total cavitation-erosion test duration of 20 h. For the mass loss of specimens prepared at different laser powers, the average value of parallel tests was taken as the mass loss for cavitation-erosion test under the corresponding laser power. The surfaces of all specimens for the above-mentioned performance tests were ground sequentially with 180#, 400#, 600#, 1000#, 1200# and 2000# grit SiC sandpapers, and further polished to a mirror finish using 3 μm and 0.05 μm polishing cloths. Mass measurements required in performance tests were carried out using a high-precision balance (Sartorius LE225D, Göttingen, Germany) with an accuracy of 0.01 mg.

## 3. Results and Discussion

### 3.1. Surface Morphology and Mechanical Properties

**Figure 1:**
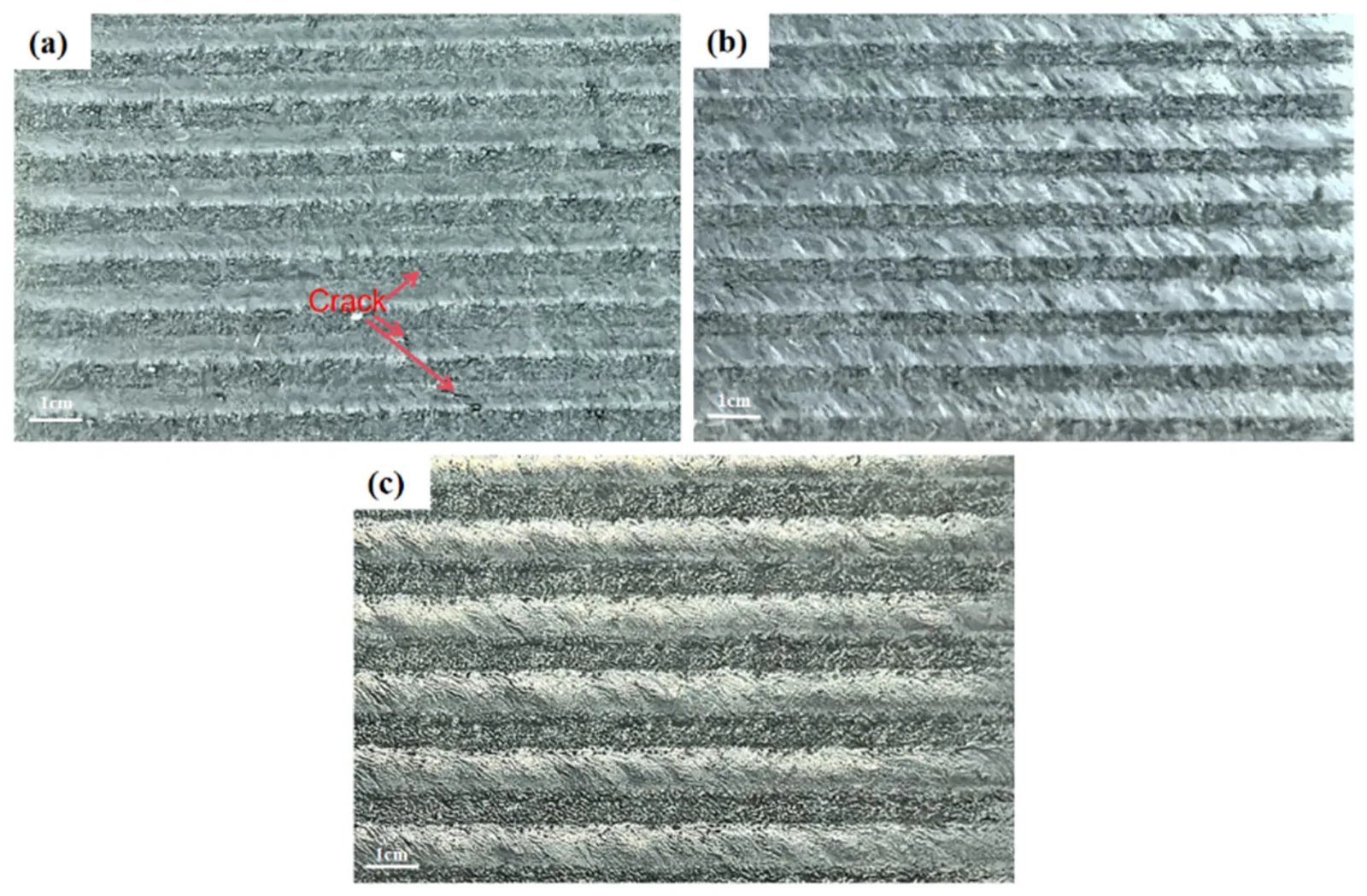
Macroscopic surface morphologies of coatings prepared by laser cladding under different laser powers: (**a**) 1000 W, (**b**) 1200 W, (**c**) 1400 W.

Figure 1 shows the macroscopic surface morphologies of coatings fabricated by laser cladding at different laser powers. Observations reveal that no cracks or pores are present on the cladding layers and no macroscopic defects can be detected when *p* > 1000 W, indicating favorable forming quality. By contrast, cracks emerge on the cladding-layer surface when *p* ≤ 1000 W, corresponding to poor forming quality.

**Figure 2:**
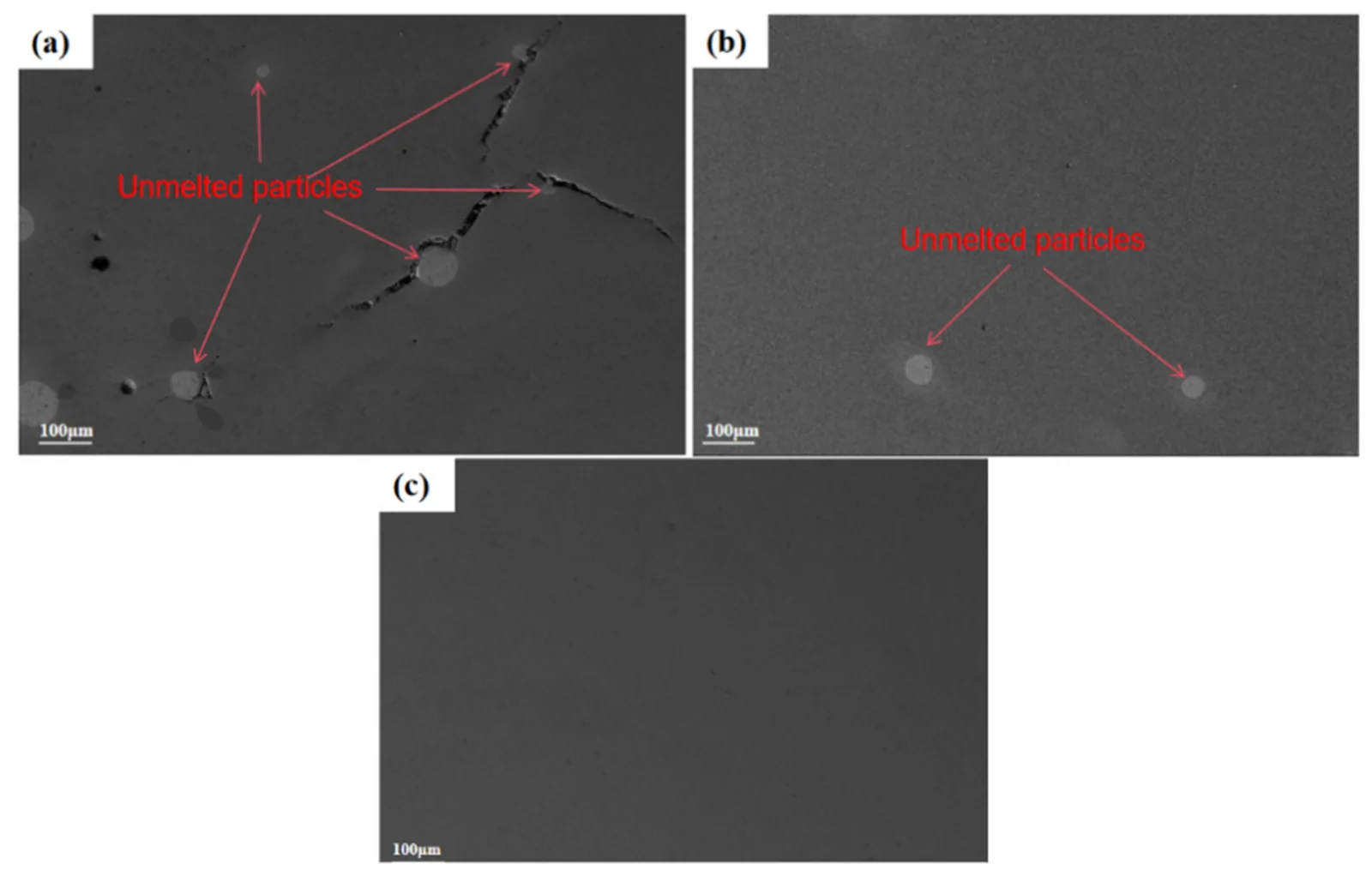
Microscopic surface morphology of coatings under different laser powers: (**a**) 1000 W, (**b**) 1200 W, (**c**) 1400 W.

Figure 2 presents the micro-surface morphologies of coating specimens fabricated at different laser powers characterized by SEM. Qualitative results can be obtained from Figure 2a–c. showing that the coating obtained at 1000 W contains a large number of unmelted refractory particles, and cracks initiate around the interfaces of these refractory particles. The coating prepared at 1200 W possesses fewer unmelted refractory particles, while no cracks initiate at particle interfaces. No unmelted particles can be found on the coating surface at 1400 W, and the surface is relatively intact and smooth. It is inferred that crack initiation may occur when the laser power is relatively low. A lower laser power corresponds to lower heat input, which leads to a low molten-pool temperature, insufficient melting and poor fluidity of the melt. The molten pool solidifies at an extremely high cooling rate, generating remarkably inhomogeneous temperature gradients both inside the cladding layer and at the coating–substrate interface, which induce large transient thermal stress and residual tensile stress. Once the residual stress exceeds the ultimate tensile strength of the cladding material, cracks initiate on the cladding surface and form visible macroscopic crack defects [28]. Another possible reason is that low laser power may cause an excessively high cooling rate, so the refractory particles contained in the cladding powder cannot be fully melted. Owing to the high cooling rate, refractory particles cannot shrink compatibly with the surrounding matrix. Severe thermal stress arises around particle interfaces, giving rise to stress concentration and serving as crack-nucleation sites.

**Figure 3:**
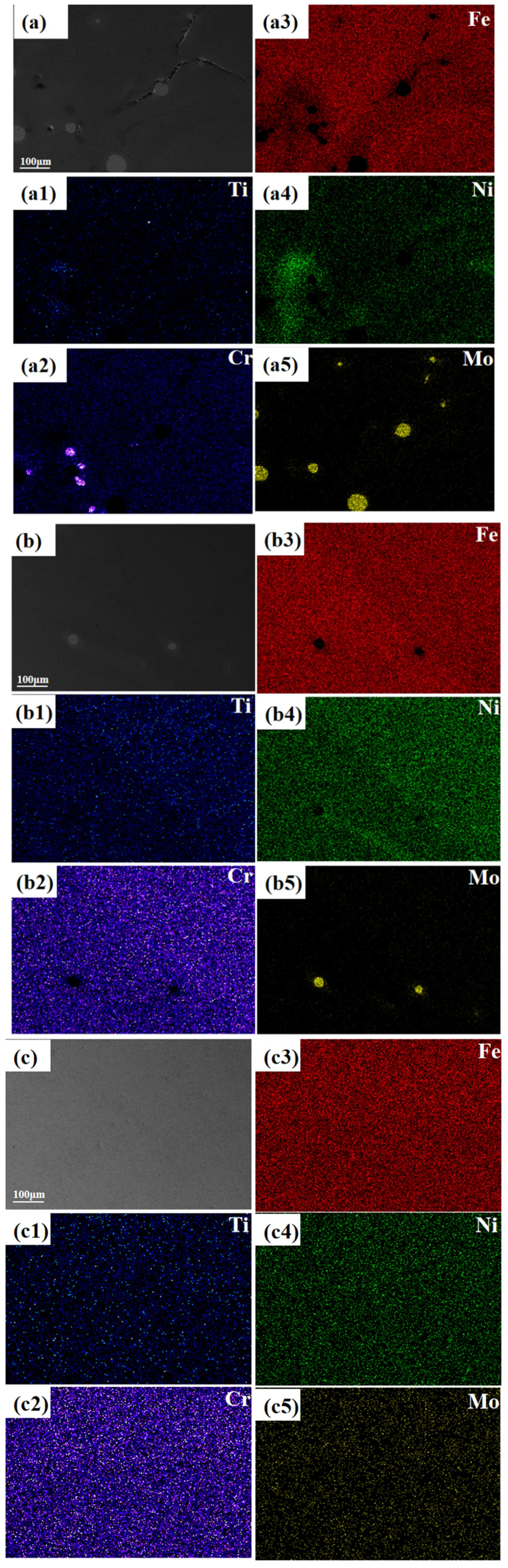
Surface EDS element maps of coatings prepared at different laser powers: (**a**) 1000 W coating, (**b**) 1200 W coating, (**c**) 1400 W coating. (**a1**–**a5**), (**b1**–**b5**), and (**c1**–**c5**) are the distribution maps of the corresponding elements, respectively.

Figure 3 illustrates the elemental distribution within coatings fabricated under different laser powers. It can be seen from Figure 3(a5) and Figure 3(b5) that the unmelted refractory particles are Mo-enriched regions. The melting points of Ni, Fe, Ti, Cr and Mo in the feedstock powder are 1455 °C, 1538 °C, 1668 °C, 1857 °C and 2623 °C, respectively. It can be further speculated from the melting points that the unmelted particles are Mo-enriched regions. By comprehensively observing the surface EDS maps of coatings prepared under three different laser powers, it can be qualitatively found that the coating at 1200 W contains fewer unmelted Mo particles and exhibits more homogeneous distribution of the other four elements compared with the 1000 W coating. All elements are relatively homogeneously distributed for the 1400 W sample versus the 1000 W sample. These results demonstrate that low laser power gives rise to a low molten-pool temperature and high cooling rate, which indeed hinder elemental diffusion. Further observation on the 1000 W coating shows local Cr enrichment. This phenomenon may lead to inhomogeneous hardness distribution and aggravate the heterogeneity of mechanical properties inside the coating. Moreover, brittle hard phases tend to form in Cr-rich zones and drastically degrade local ductility. Combined with the stress-concentration effect induced by Mo particles, the tendency for crack initiation and propagation in the coating is remarkably enhanced.

**Figure 4:**
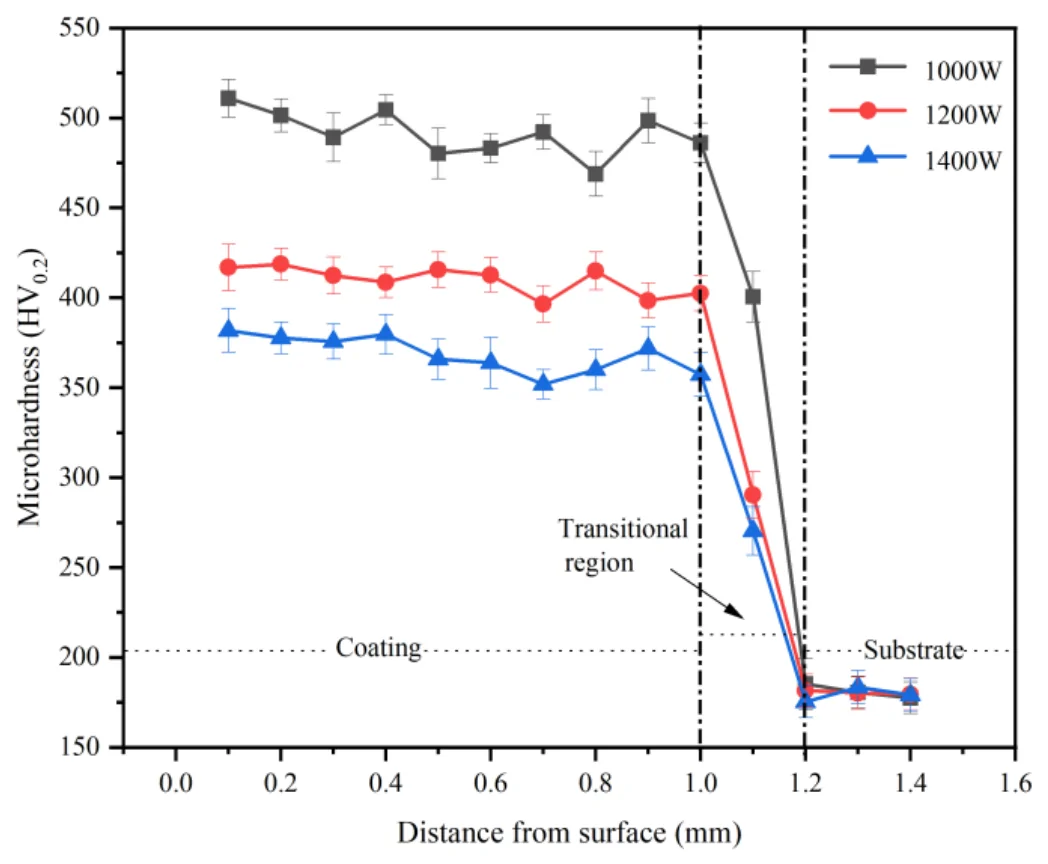
Cross-sectional hardness of coatings produced at different laser powers.

Figure 4 shows the cross-sectional hardness profiles of coatings under different laser powers measured by a micro-Vickers hardness tester. It can be seen that the coating hardness decreases with the increase in laser power. The average hardness values of the coatings prepared at 1000 W, 1200 W and 1400 W are 485.67 HV_0.2_, 402.96 HV_0.2_ and 363.15 HV_0.2_, respectively. This phenomenon can first be attributed to the fact that Cr enrichment may promote the formation of Cr-rich hard regions or intermetallic phases, which increases the hardness of the coating [29].

**Figure 5:**
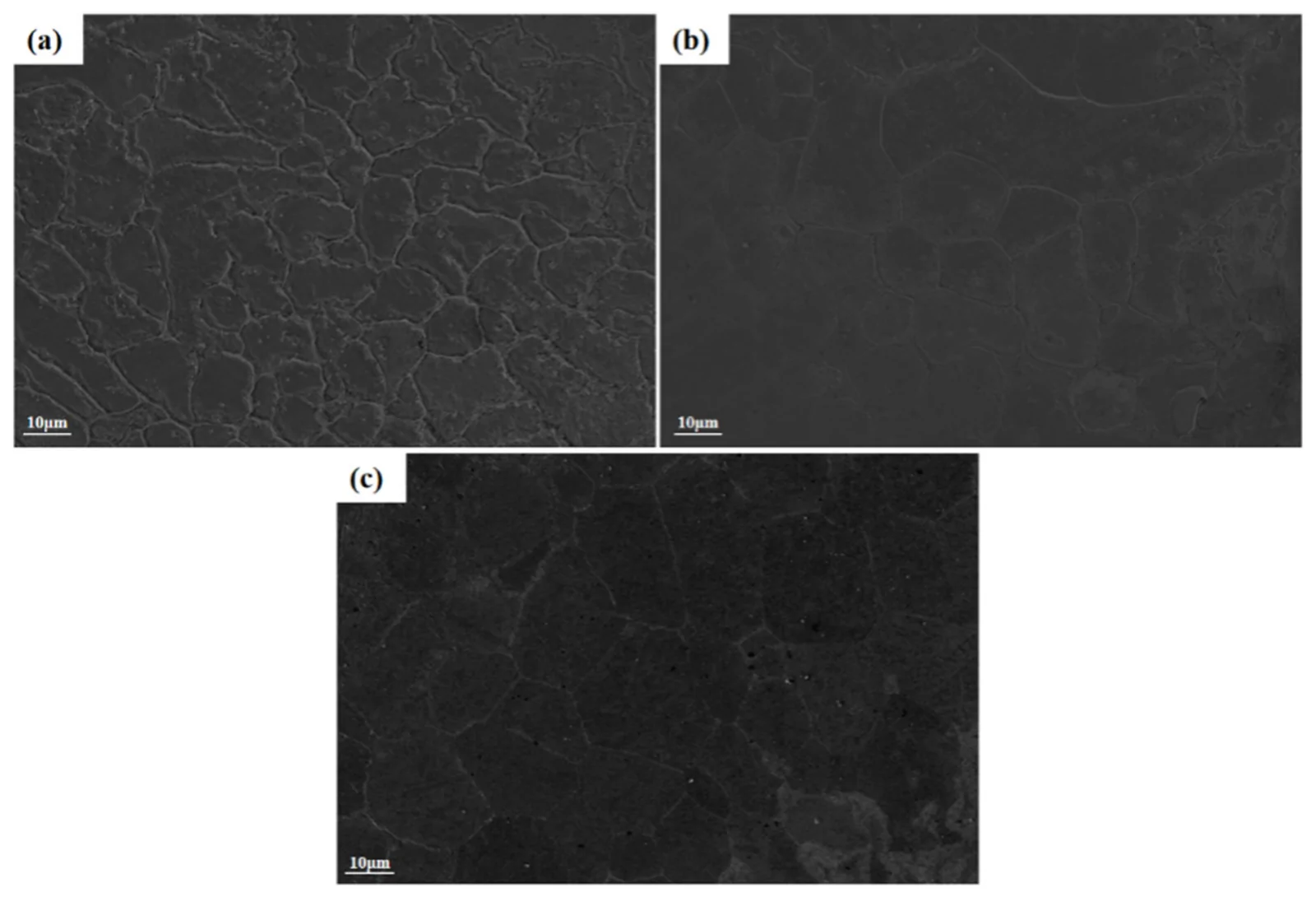
Surface grain size images of coatings produced at different laser powers: (**a**) 1000 W coating, (**b**) 1200 W coating, (**c**) 1400 W coating.

It can be qualitatively inferred from Figure 5 that the overall grain size on the coating surface tends to increase gradually with rising laser power. Therefore, the hardness difference among coatings fabricated at three laser powers can also be explained by the fact that a lower laser power leads to a higher cooling rate and faster solidification nucleation rate, resulting in smaller grain sizes, which is inferred to produce a grain refinement strengthening effect. As laser power increases, grain coarsening occurs, leading to a decrease in hardness.

**Figure 6:**
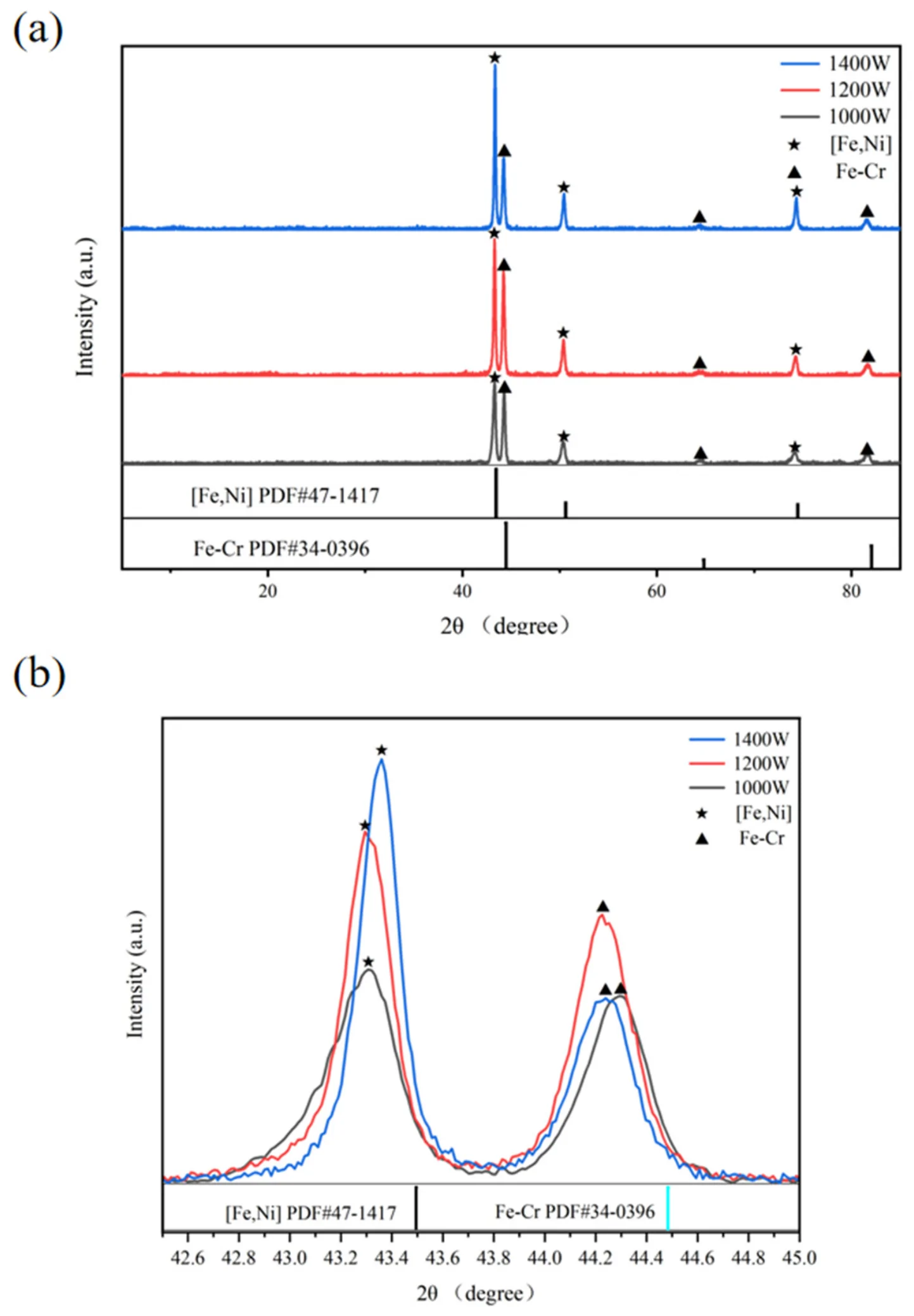
(**a**) XRD scanning patterns of the coatings, (**b**) partial enlarged view of XRD scanning patterns of the coatings.

Figure 6 presents the XRD patterns obtained from X-ray diffraction scans of coatings fabricated at three different laser powers. It can be found from Figure 6a that under different laser powers, the main phases in the coatings are the [Fe,Ni] solid-solution phase with FCC structure dominated by Fe, Cr and Ni elements and the Fe-Cr solid-solution phase with BCC structure. This indicates that the variation in laser power does not alter the fundamental phase types of the coatings, and no substantial new intermetallic compound phases are generated. As shown in Figure 6b, the diffraction peaks of the two phases shift toward lower angles for all three coatings. This is according to Bragg’s law: 2dsin θ = nλ, where d is the interplanar spacing, θ is the angle between incident X-ray beam and crystal plane, n is the order of reflection, and λ is the wavelength of X-ray. A decrease in diffraction angle θ corresponds to an increase in interplanar spacing d, indicating that the lattice spacing of all coatings increases. This feature is speculated to originate from the typical non-equilibrium rapid solidification effect of laser cladding. Ti and Mo with large atomic radii cannot precipitate via equilibrium diffusion and are forcibly dissolved into [Fe,Ni] and Fe-Cr solid solutions, stretching the crystal lattice and increasing the lattice spacing [30].

As shown in Figure 6b, the shift in diffraction angle θ for the [Fe,Ni] phase gradually decreases with increasing laser power. Combined with Bragg’s law, it can be concluded that the increment of lattice spacing of this phase gradually declines as the laser power increases. Observation on the diffraction-peak intensities of the two phases further reveals that the peak intensity of the [Fe,Ni] phase increases with rising laser power. This may be attributed to the ultra-fast solidification at 1000 W, which induces high-density dislocations, lattice distortion and micro-residual stress. The long-range order of crystal lattice is severely deteriorated, which manifests as broadened and remarkably weakened diffraction peaks in XRD patterns [31].

### 3.2. Wear Resistance Performance

**Figure 7:**
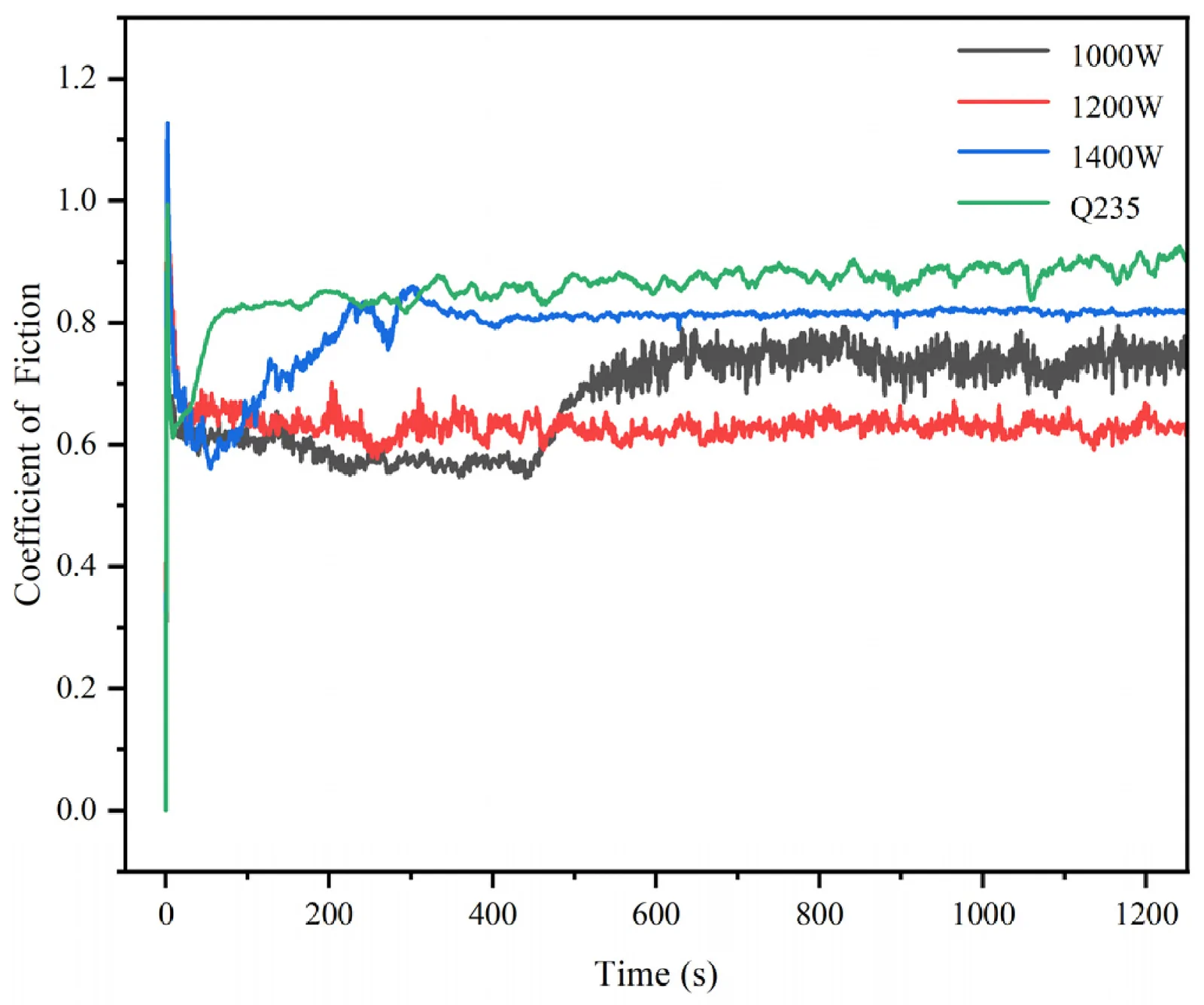
Friction–wear curves of Q235 steel and coatings fabricated under different laser powers.

Figure 7 depicts the curve representing the variation in the friction coefficient with time during the friction-and-wear test on the coatings produced under different laser power conditions and on the surface of Q235 steel. In the initial stage, the friction coefficient of all specimens decreases. Friction leads to a temperature rise of the friction pair, which may promote the elements contained in the coating to combine with O and form an oxide film with self-lubricating properties [32]. These oxides reduce the shear stress between friction pairs and thus lower the friction coefficient [32]. Nevertheless, the real contact area expands upon continuous friction, resulting in a gradual increase in friction coefficient until it reaches a stable state.

**Figure 8:**
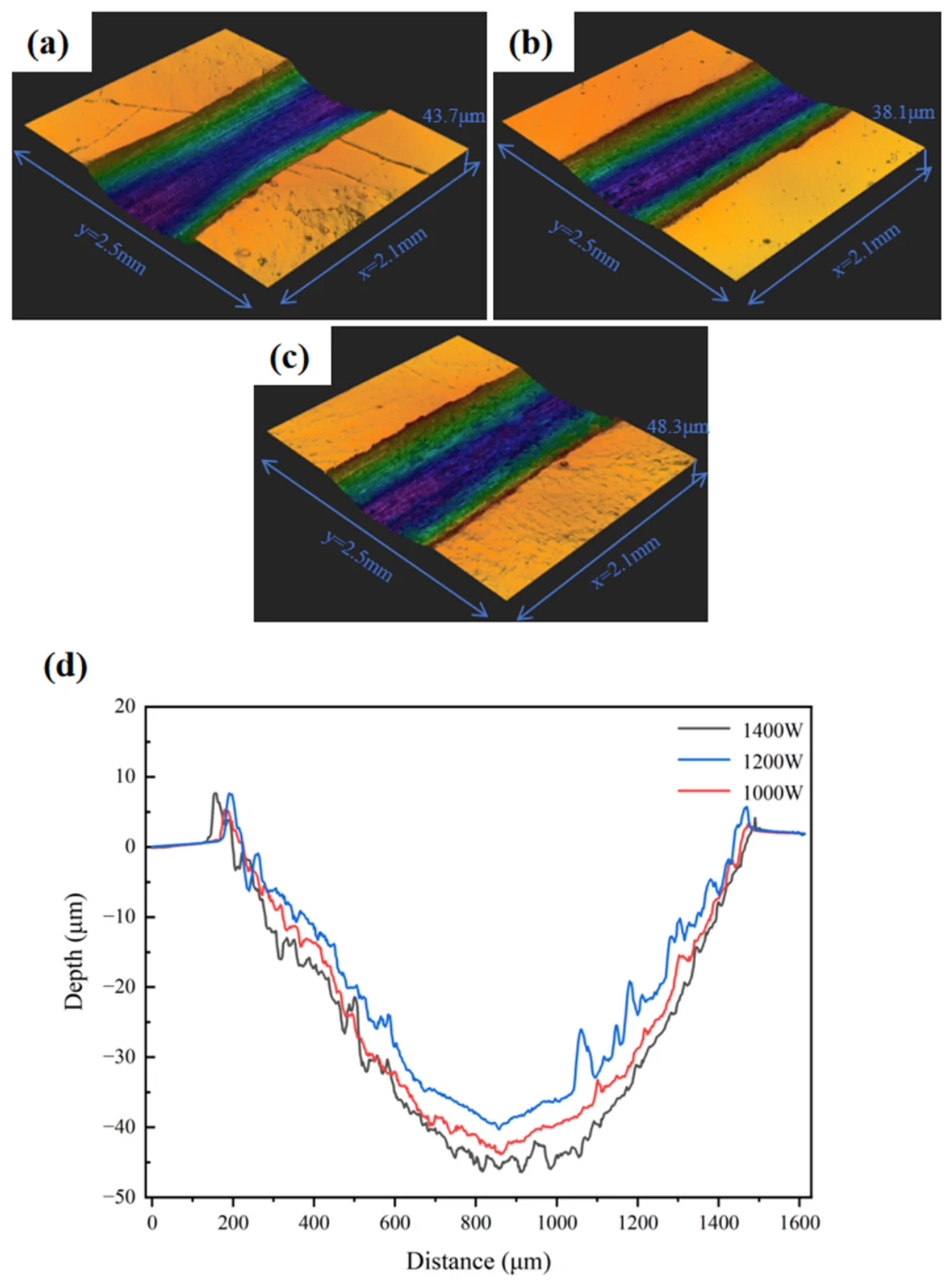
Three-dimensional and two-dimensional wear-track morphologies of coatings fabricated at different laser powers: (**a**) 1000 W coating, (**b**) 1200 W coating, (**c**) 1400 W coating, (**d**) two-dimensional wear-track morphology.

Figure 8 shows the three-dimensional and two-dimensional surface morphologies of coatings fabricated at different laser powers after friction–wear tests. The furrow traces of all coatings are inhomogeneous and aligned with the friction direction. Under the present test conditions, at the same sliding distance, the coating fabricated at 1200 W with well-matched hard and soft phases exhibits superior wear resistance compared with the other two coatings, and its wear depth is also relatively smaller. The wear-rate calculation formula for coatings is expressed as *W* = *V*_loss_/(*F*_N_·*L*), where W is the wear rate, V_loss_ is the wear volume, F_N_ is the applied normal load, and L is the sliding distance. Under the applied load of 20 N and friction duration of 30 min, the sliding distance was 10 mm. The experimentally calculated wear rates of the three coatings and Q235 steel are listed in Table 3. As shown in Table 3, under the test conditions, the coating prepared at 1200 W exhibits a relatively lower wear rate among the three coatings. Based on the friction coefficient and wear rate, it can be found that the coating fabricated at 1200 W has superior wear resistance among the three coatings. The wear rates of all three coatings decrease to varying degrees compared with that of Q235 steel.

**Figure 9:**
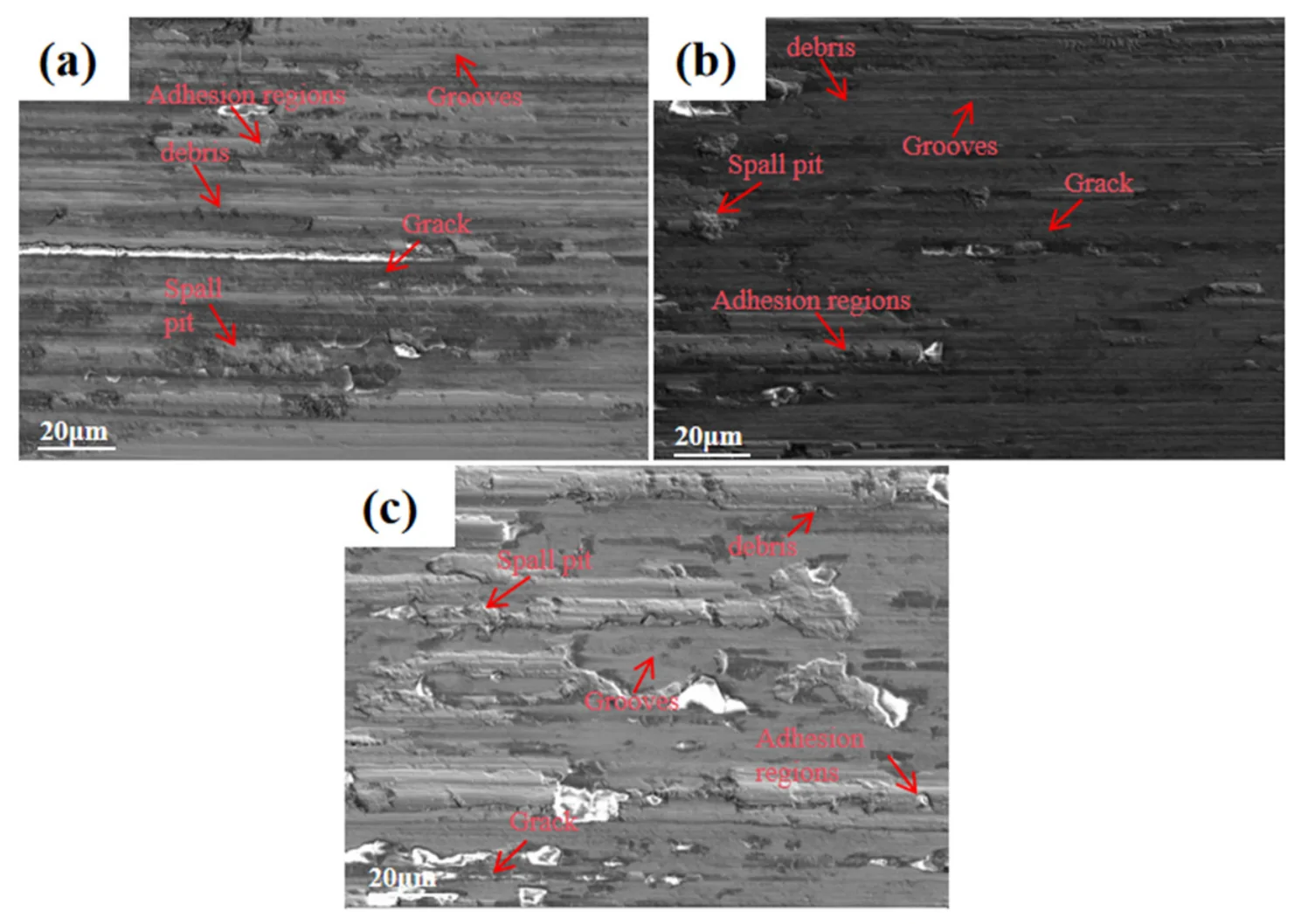
Surface micro-morphologies of friction and wear of coatings fabricated at different laser powers: (**a**) coating at 1000 W, (**b**) coating at 1200 W, (**c**) coating at 1400 W.

Wear resistance is not governed solely by microhardness, but results from the combined effects of hardness, microstructure homogeneity, defect characteristics and two-phase microstructures. Figure 9 presents the micro-morphologies of coatings prepared at different laser powers after friction–wear tests. The 1000 W coating exhibits the highest microhardness, which theoretically provides effective resistance against abrasive plowing. Nevertheless, unmelted refractory particles, local Cr segregation and abundant microcrack defects exist under this processing condition. Under cyclic shear stress during friction, brittle spalling occurs on the surface layer and numerous spalling pits are formed, which deteriorates the wear resistance. The dominant wear mechanisms are abrasive wear and brittle spalling. Abrasive particles and parallel plow grooves appear on the worn surface. At a laser power of 1200 W, sufficient heat input may promote the full alloying of abundant Mo, eliminate intrinsic crack sources inside the coating, and achieve more homogeneous Cr distribution. Moreover, the cooling rate may be moderate at this laser power, achieving a balance between strengthening effect and stress release. The [Fe,Ni] and Fe-Cr solid-solution phases possess favorable crystallographic integrity, and the hard–soft two phases are well matched. Consequently, the coating exhibits superior wear resistance compared with the other two coatings. The dominant wear mechanism of the coating at this laser power is abrasive wear. As shown in Figure 9, the coating fabricated at this laser power exhibits shallow plow grooves and relatively few spalling pits after the friction-and-wear test. When the laser power further increases to 1400 W, excessively long high-temperature holding time may lead to obvious grain coarsening, which reduces the overall coating hardness. Severe plastic deformation takes place on the surface during friction. The dominant wear mechanisms of the coating at this laser power are abrasive wear and adhesive wear. As shown in Figure 9, large-area severe spalling and adhesive wear occur during the friction-and-wear process.

**Figure 10:**
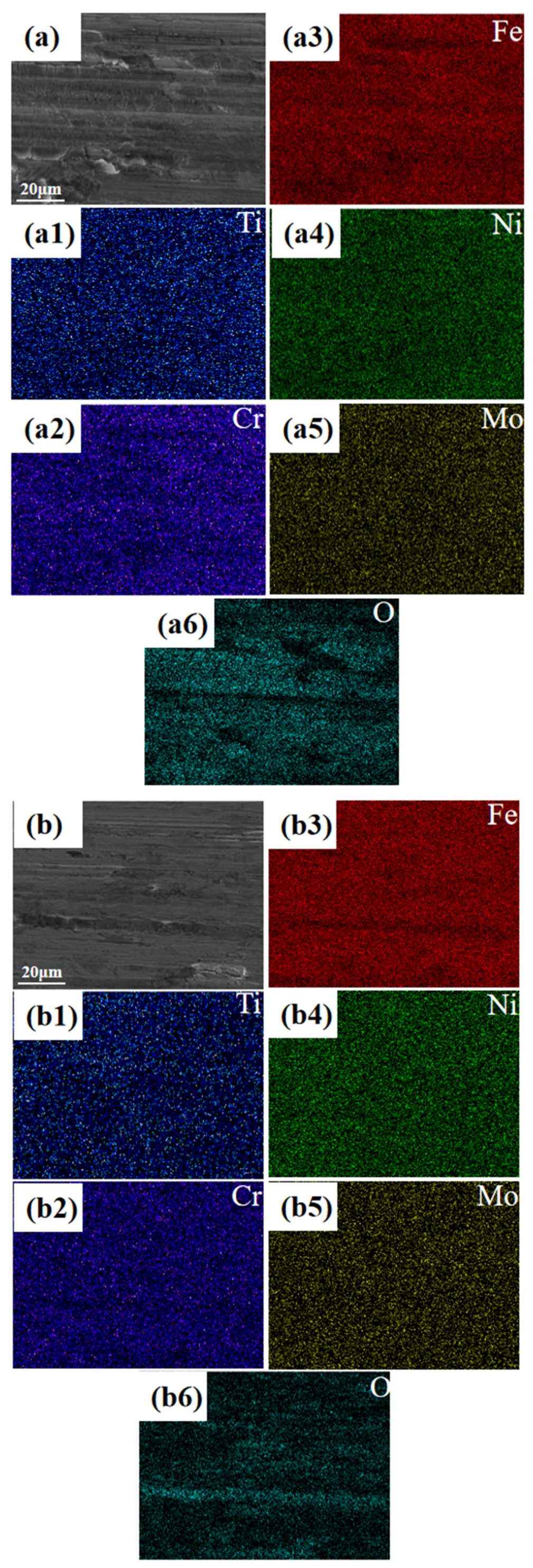

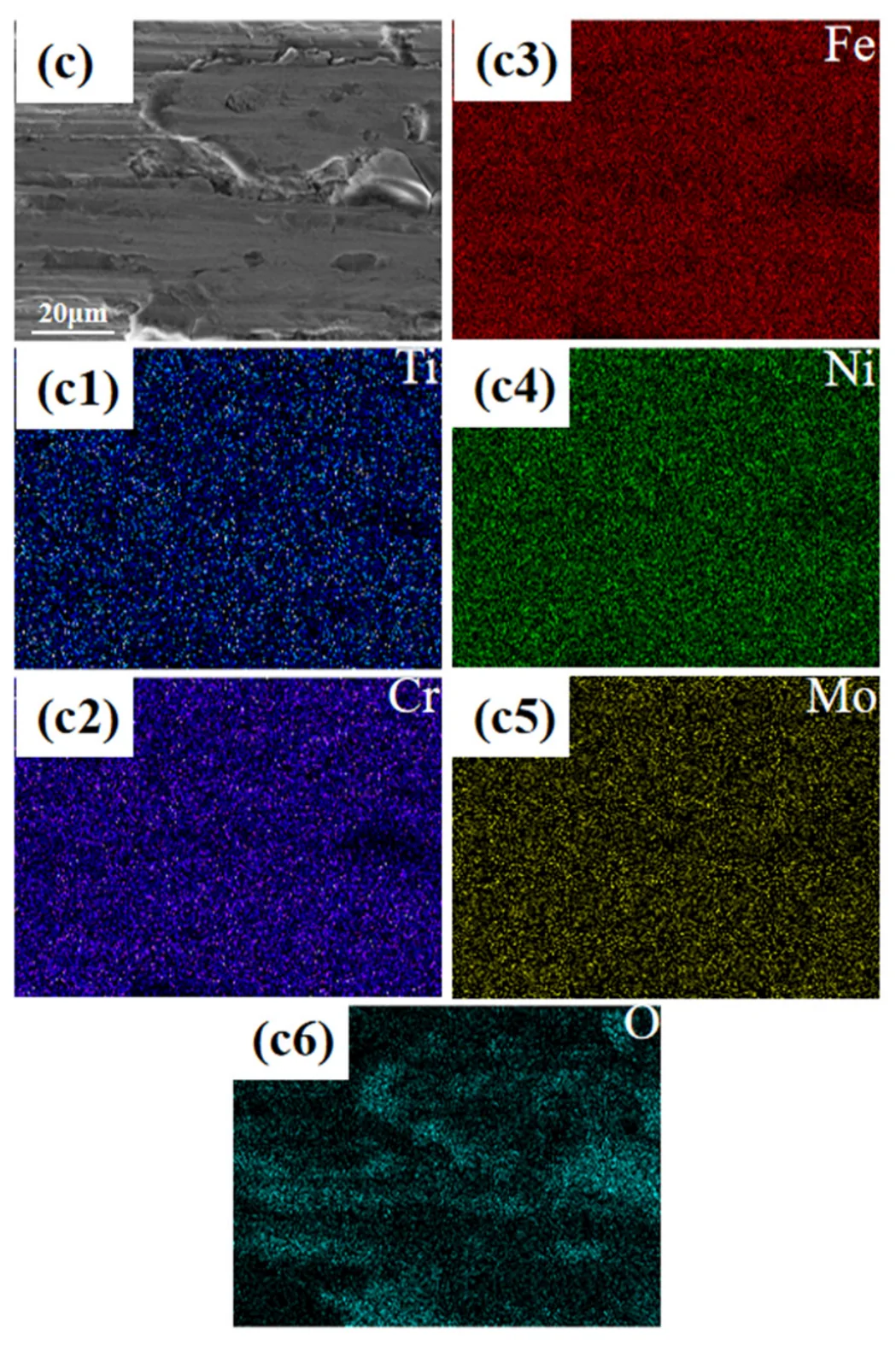
EDS elemental maps of the worn track surfaces of coatings fabricated at different laser powers: (**a**) coating at 1000 W, (**b**) coating at 1200 W, (**c**) coating at 1400 W. (**a1**–**a6**,**b1**–**b6**,**c1**–**c6**) are the corresponding element distribution maps.

Figure 10 illustrates the elemental distribution of the three coatings after friction–wear tests. It can be analyzed from the maps that oxygen is detected in all three coatings after friction–wear testing. Heat is generated during reciprocating friction of friction pairs, which triggers oxidation on the worn surface and forms an oxide film covering the wear scar of the coating. This oxide film may be a brittle oxide containing Fe, Cr, Ni, Ti and Mo elements. Microcracks and fracture occur once the applied stress exceeds the bonding strength of the oxide film. With crack propagation, the oxide film peels off in sheets and forms oxide wear debris. Fresh alloy coating is exposed in the spalled region and undergoes re-oxidation. Such cyclic processes induce oxidative wear and accelerate the deterioration of wear resistance. Observations from Figure 10 reveal that coatings prepared at 1000 W and 1400 W suffer from more severe oxidative wear compared with the 1200 W coating. This also demonstrates that among the three laser powers investigated in this work, the coating fabricated at 1200 W exhibits relatively superior wear resistance.

### 3.3. Cavitation-Erosion Resistance

**Figure 11:**
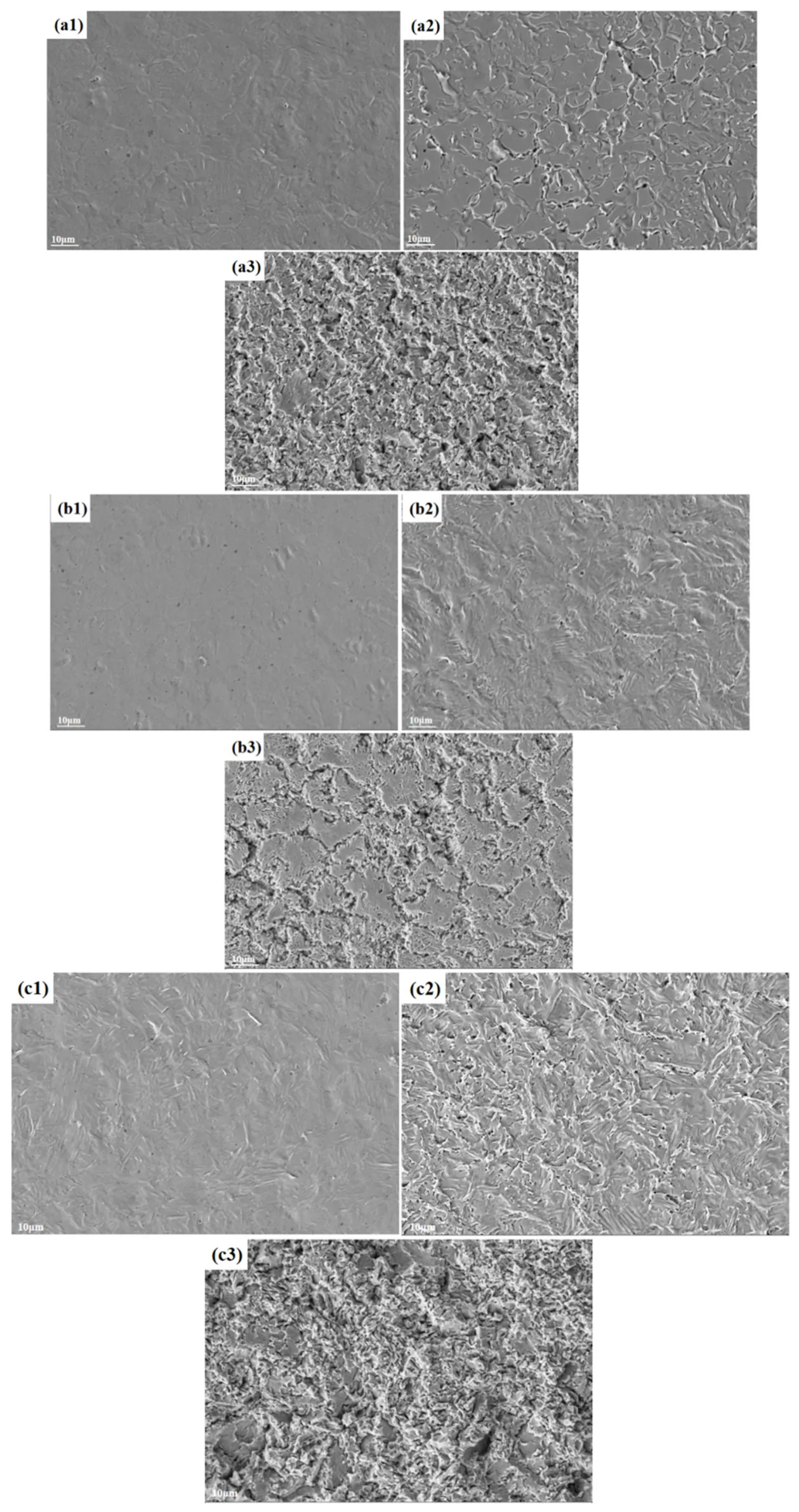
Surface micro-morphologies of coatings fabricated at different laser powers after cavitation erosion for 1 h, 4 h and 20 h: (**a1**–**a3**) coating at 1000 W, (**b1**–**b3**) coating at 1200 W, (**c1**–**c3**) coating at 1400 W.

Figure 11 shows the micro-morphologies of coatings fabricated at laser powers of 1000 W, 1200 W and 1400 W after cavitation-erosion testing for 1 h, 4 h and 20 h. It can be observed from Figure 11(a1–a3) that the coating prepared at 1000 W remains relatively intact after 1 h cavitation erosion, with only faint plastic-deformation traces and no obvious pits or spalling. The high hardness enables the coating to resist impact loading within a short period. After 4 h of cavitation erosion, surface damage is remarkably aggravated. Numerous network-like microcracks initiate, and small-scale spalling occurs in local regions. This is speculated to arise from stress concentration around brittle hard phases induced by local Cr segregation and abundant unmelted hard particles. These sites act as crack sources. Such hard brittle phases and particles bring about microstructural inhomogeneity, which facilitates the formation of network-like cracks under cyclic loading. After 20 h cavitation erosion, the coating surface becomes rugged. Network-like cracks continuously propagate and interconnect, leading to sheet-like spalling and large spalling pits, which induce severe cavitation-erosion damage. As shown in Figure 11(b1–b3), the coating fabricated at 1200 W possesses a relatively smooth surface after 1 h cavitation erosion. Only slight plastic-flow traces are observed, and nearly no cavitation pits or microcracks can be detected. After 4 h cavitation erosion, only a small number of dispersed shallow plastic pits are generated with scarce cracks, and no interconnected network cracks are formed. It is inferred that the homogeneous two-phase microstructure may restrain crack initiation and growth, resulting in slow damage evolution. Certain spalling and pits emerge after 20 h cavitation erosion. Nevertheless, compared with the 1000 W and 1400 W coatings, it exhibits smaller spalled areas and shallower pits without large-scale contiguous lamellar peeling. From Figure 11(c1–c3), abundant plastic-slip traces are observed on the 1400 W coating after 1 h cavitation erosion. Excessive heat input results in grain coarsening and deteriorated solid-solution strengthening. The reduced hardness weakens the resistance against plastic deformation induced by bubble micro-jet impact, and plastic damage readily accumulates on the surface layer. After 4 h cavitation erosion, plenty of pits form on the surface and local lamellar peeling takes place. After 20 h cavitation erosion, severe lamellar spalling occurs, accompanied by extensive rugged damage. Deep and dense spalling pits are produced, which cause serious material loss of the coating.

**Figure 12:**
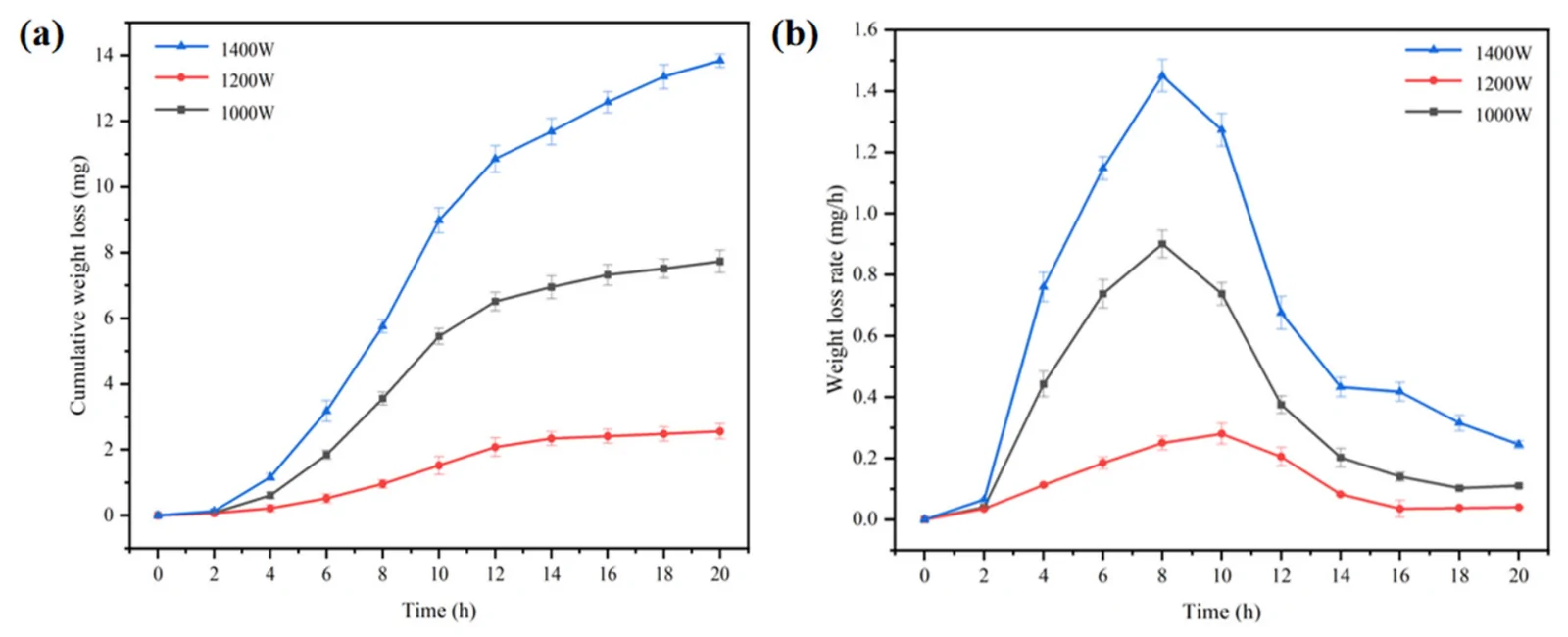
Curves of average mass loss and average mass-loss rate at different cavitation erosion times for coatings fabricated under different laser powers: (**a**) average mass loss of coatings, (**b**) average mass-loss rate of coatings.

Figure 12a,b show the curves of average mass loss and average mass-loss rate at different exposure times for the coatings fabricated at laser powers of 1000 W, 1200 W and 1400 W after 20 h of cavitation erosion, respectively. The error bars in the figure are calculated from the standard deviation of the data, representing the data dispersion. As observed from Figure 12a,b, the cumulative mass loss of all three specimens remains low and the mass-loss rate is nearly zero during the 0–2 h cavitation incubation stage. This is attributed to the fact that bubble-collapse impact mainly induces plastic deformation of the surface layer with negligible material spalling. With prolonged cavitation-erosion duration, the coatings enter the accelerated-damage stage. The 1400 W coating exhibits the fastest increase in cumulative mass loss and the largest total mass loss after 20 h, followed by the 1000 W coating, while the 1200 W coating maintains the lowest cumulative mass loss throughout the test. The instantaneous mass-loss-rate curves reveal typical cavitation-erosion kinetic characteristics for all three coatings, including the incubation stage, acceleration stage, deceleration stage and attenuation–stabilization stage. The 1400 W specimen shows the fastest rising rate and the highest peak mass-loss rate, followed by the 1000 W specimen, whereas the 1200 W specimen presents the lowest peak value. The quantitative mass-loss results are in good agreement with post-cavitation SEM observations. This result indicates that the 1200 W coating maintains a good balance between hardness and microstructural integrity due to its homogeneous microstructure and fewer metallurgical weak interfaces, and exhibits superior cavitation-erosion resistance compared with the other two coatings. As listed in Table 4, after 20 h cavitation erosion, the average mass losses of coatings prepared at 1000 W, 1200 W and 1400 W are 7.73 mg, 2.56 mg and 13.84 mg, respectively. The coating at 1200 W possesses superior cavitation-erosion resistance compared with the other two coatings. All three coatings exhibit greatly improved cavitation-erosion resistance compared with Q235 steel, indicating that laser cladding achieves remarkable performance in surface modification.

## 4. Conclusions

(1) With the increase in laser power, the hardness of the coating gradually decreases from a maximum value of 485.67 HV_0.2_ to 363.15 HV_0.2_. The unmelted particles in the coating gradually decrease, and the surface structure becomes more intact and smooth.
(2) Under the test conditions, the Fe_45_Cr_25_Ni_20_Ti_5_Mo_5_ coating fabricated at a laser power of 1200 W exhibits superior wear resistance compared with the coatings prepared at the other two laser powers. Under different laser powers, the dominant wear mechanisms of the coatings are as follows: the coating at 1000 W is dominated by abrasive wear and brittle spalling; the coating at 1200 W is mainly subjected to abrasive wear; and the coating at 1400 W is dominated by abrasive wear and adhesive wear. Oxidative wear occurs to varying degrees in all three coatings. After the 30 min friction-and-wear test under an applied load of 20 N, it is found that the wear rates of the three coatings are lower than that of Q235 steel, and the wear resistance of all three coatings is improved relative to Q235 steel.
(3) Under the test conditions, the Fe_45_Cr_25_Ni_20_Ti_5_Mo_5_ coating fabricated at the laser power of 1200 W also shows superior cavitation-erosion resistance compared with the other two coatings. After 20 h of cavitation-erosion testing, the mass loss of the coating at this power is only 2.56 mg, while that of Q235 steel reaches 82.94 mg. The coatings prepared at laser powers of 1000 W and 1400 W exhibit mass losses of only 7.73 mg and 13.84 mg, respectively. Their cavitation-erosion resistance is also greatly improved compared with Q235 steel, demonstrating that multi-principal-element alloy coatings prepared by laser cladding achieve an excellent effect in improving the surface cavitation-erosion resistance of materials.

## Figures and Tables

**Table 1:** Powder composition ratio.

**Name of spherical powder**	Fe	Cr	Ni	Ti	Mo
**Proportion of each powder (at.%)**	45	25	20	5	5

**Table 2:** Process parameters.

Sample	Laser Power (W)	Powder Feeding Rate (g/min)	Scan Rate (mm/s)
S1	1000 W	6	5
S2	1200 W	6	5
S3	1400 W	6	5

**Table 3:** Wear rates of coatings and Q235 steel prepared under different laser powers.

Sample	20 N
Coatings made of 1000 W	3.92 × 10^−5^ mm^3^/N∙m
Coatings made of 1200 W	3.15 × 10^−5^ mm^3^/N∙m
Coatings made of 1400 W	4.37 × 10^−5^ mm^3^/N∙m
Q235 steel	5.18 × 10^−5^ mm^3^/N∙m

**Table 4:** Average mass loss of each coating and Q235 steel after 20 h of cavitation erosion.

Sample	Average Mass Loss (mg)
Coatings made of 1000 W	7.73
Coatings made of 1200 W	2.56
Coatings made of 1400 W	13.84
Q235 steel	82.94

## Data Availability

The original contributions presented in this study are included in the article. Further inquiries can be directed to the corresponding authors.
